# The Puzzle of the Regioselectivity and Molecular Mechanism of the (3+2) Cycloaddition Reaction Between E-2-(Trimethylsilyl)-1-Nitroethene and Arylonitrile *N*-Oxides: Molecular Electron Density Theory (MEDT) Quantumchemical Study

**DOI:** 10.3390/molecules30040974

**Published:** 2025-02-19

**Authors:** Mikołaj Sadowski, Ewa Dresler, Radomir Jasiński

**Affiliations:** 1 Department of Organic Chemistry and Technology, Cracow University of Technology, Warszawska 24, 31-155 Kraków, Poland; mikolaj.sadowski@doktorant.pk.edu.pl; 2 Łukasiewicz Research Network—Institute of Heavy Organic Synthesis “Blachownia”, Energetyków 9, 47-225 Kędzierzyn-Koźle, Poland; ewa.dresler@icso.lukasiewicz.gov.pl

**Keywords:** (3+2) cycloaddition, nitrile *N*-oxides, mechanism, DFT, MEDT

## Abstract

The regioselectivity and molecular mechanism of the (3+2) cycloaddition reaction between E-2-(trimethylsilyl)-1-nitroethene and arylonitrile *N*-oxides were explored on the basis of the *ω*B97XD/6-311+G(d) (PCM) quantumchemical calculations. It was found that the earlier postulate regarding the regioselectivity of the cycloaddition stage should be undermined. Within our research, several aspects of the title reaction were also examined: interactions between reagents, electronic structures of alkenes and nitrile oxides, the nature of transition states, the influence of the polarity solvent on the reaction selectivity and mechanism, substituent effects, etc. The obtained results offer a general conclusion for all of the important aspects of some groups of cycloaddition processes.

## 1. Introduction

Isoxazoles (1,2-oxazoles) are important heterocyclic compounds characterized by a wide range of potential applications in industry, pharmacy, biotechnology, and other areas [1,2,3,4,5,6]. These types of heterorganic compounds evidently exhibit antibacterial, antibiotic, anticancer, antifungal, and antitumor, among others, bioactivities. In agronomy, analogs of the isoxazole are applied as insecticides, herbicides, and components that stimulated the antiparasitic effect for animals and plants. Additional applications are connected with the possibility of the conversion of the heterocyclic ring to enaminoketones, γ-amino alcohols, α,β-unsaturated oximes, β-hydroxy nitriles, and other compounds [7,8,9,10,11].

**Scheme 1:**
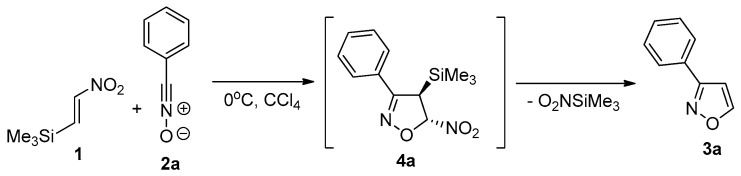
Experimentally observed course of the (3+2) cycloaddition reaction between E-2-(trimethylsilyl)-1-nitroethene (**1**) and benzonitrile *N*-oxide (**2a**).

The preparation of isoxazole heterocyclic systems is possible via different types of preparative protocols, such as the acylation of oximes [12], the condensation of hydroxylamines with carbonyl compounds [13], or the cyclisation of alkynes functionalized by oxime moiety [14]. The most universal approach is, however, the (3+2) cycloaddition (32CA) reaction with the participation of nitrile *N*-oxides as three atom components (TACs) [15,16]. It is generally known that the acetylenic components are less reactive in comparison to analogous ethene derivatives [17,18,19]. Therefore, an optimal strategy for the preparation of isoxazoles is 32CA processes between nitrile *N*-oxides and the further aromatization of primary obtained 2-isoxazolines. This is possible, for example, via an easy dehydronitration scheme [20]. *Padwa* and coworkers described these types of transformations with the participation of the benzonitrile *N*-oxide (**2a**) and E-2-(trimethylsilyl)-1-nitroethene (**1**) [21,22]. In the course of this process, the denitrative aromatization of the primary nitroisoxazoline (**4a**) is realized spontaneously, yielding 3-phenylisoxazole (**3a**) as the final product (Scheme 1).

**Scheme 2:**
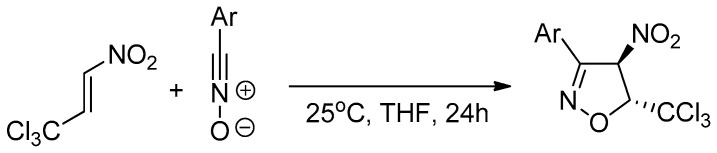
Experimentally observed course of the (3+2) cycloaddition reaction between E-3,3,3-trichloro-1-nitroprop-1-ene and arylonitrile *N*-oxides.

It is interesting that the authors proposed 3-phenyl-4-trimethylsillyl-5-nitroisoxazoline (**4a**) as the reaction intermediate. The existence of this cycloadduct in the reaction mixture was, however, not confirmed by any method. On the other hand, analogous 32CAs with the participation of other 2-R-nitroethenes are realized with a completely different regioselectivity. For example, the 32CAs between arylonitrile *N*-oxides and E-3,3,3-trichloro-1-nitroprop-1-ene lead to respective 3-aryl-4-nitro-5-trichloromethyl-2-isoxazolines as single reaction products (Scheme 2) [23]. This is a consequence of the electrophilic activation of the two-position of the nitrovinyl moiety. This effect was very recently observed regarding different 32CAs with the participation of conjugated nitroalkenes [24,25,26,27,28].

**Scheme 3:**
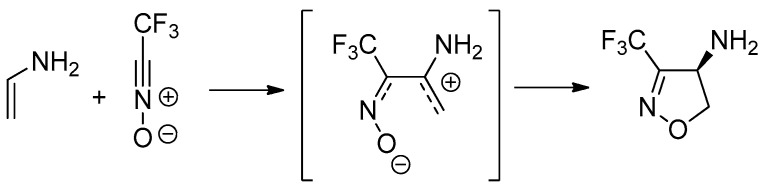
The stepwise zwitterionic mechanism of the 32CA between trifluroacetonitrile *N*-oxide and vinylamine.

So, the real regioselectivity of the title reaction should be a treatment that is unknown at this moment. Additionally, the mechanistic background of this cycloaddition also requires a respective exploration. According to the actual state of knowledge, the earlier theory about the “concerted” nature of 32CA independently of the nature of addends completely failed [29]. At this moment, different types of mechanisms can be considered: non-polar (synchronous, asynchronous, biradical stepwise) or polar (synchronous, asynchronous one-step two-stage, zwitterionic stepwise). For example, via the stepwise zwitterionic mechanism, the (3+2) cycloaddition between trifluroacetonitrile *N*-oxide and vinylamine is realized (Scheme 3) [30,31].

**Scheme 4:**
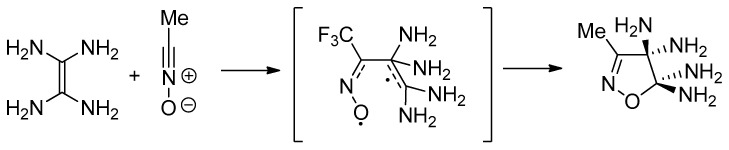
The stepwise biradical mechanism of the 32CA between acetonitrile *N*-oxide and tetraaminoethene.

On the other hand, the (3+2) cycloaddition between acetonitrile *N*-oxide and tetraaminoethene proceeds according to the stepwise mechanism with the intervention of the biradical intermediate [32] (Scheme 4).

**Scheme 5:**
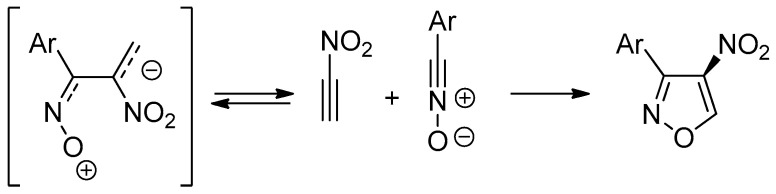
The formation of the zwitterionic intermediate in the reaction between nitroacetylene and arylonitrile *N*-oxides.

Lastly, in some cases, the formation of acyclic intermediates characterized by “extended” conformation can compete with the formation of (3+2) cycloadducts via the single-step cycloaddition method [33] (Scheme 5).

**Scheme 6:**
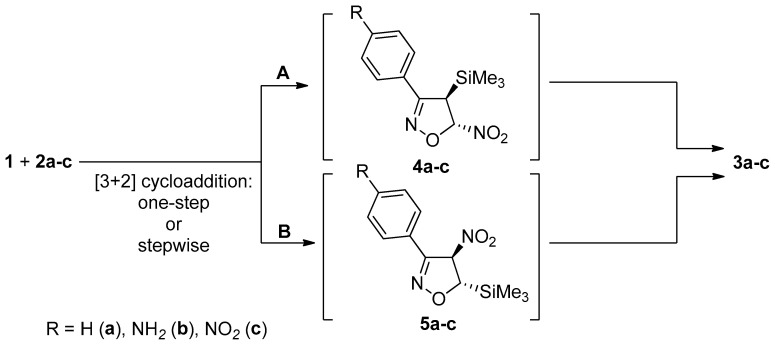
Theoretical possibility of the regioisomeric paths of the (3+2) cycloaddition reaction between E-2-(trimethylsilyl)-1-nitroethene (**1**) and benzonitrile *N*-oxide (**2a**).

Due to the issues mentioned above, we performed a comprehensive quantumchemical study in the framework of the Molecular Electron Density Theory approach [34,35,36]. In particular, we decided to (i) analyze the global and local reactivity of the reaction components based on the CDT tools [37,38]; (ii) fully explore both regioisomeric reaction paths (Scheme 6). In the framework of the second area of this research, we analyzed all of the detected critical structures, including the influence of the substituent effect as well as the polarity of the solvent on the reaction course. In addition, at the same level of theory, we examined an analogous reaction with the participation of 1-acetyl-1-trimethylsyllil-ethene (**7**) because the regioselectivity of this process was confirmed experimentally.

## 2. Results and Discussion

### Electronic Interactions

Substrates **1** and **2a**–**c** were firstly characterized by the CDFT approach [38] substrates, and computations were performed using Gaussian 16 review C.01 at the *ω*B97XD/6-311G(d) theory level. For the substrates optimized structures, the ELF function was computed using Topmod 09 software and visualized with paraview.

The three atom components (TACs) in the studied reaction are N-nitrile oxides (**2a**–**c**), and the alkene component is ethene (**1**). As an anchor point to compare the silyl analogue, we have also computed all the parameters for (*E*)-3,3-dimethyl-1-nitrobut-1-ene (**6**).

The global electrophilicity and nucleophilicity of the substrates are presented in Table 1.

Electrophilicity and nucleophilicity can be qualified according to the electrophilicity–nucleophilicity scale proposed and successfully applied by Domingo et al. [37], where qualification allows for a quick mental assessment of the compounds’ reactivity. The original intervals for each category were proposed for the results obtained via B3LYP/6-31G(d) model. As *ω*B97XD/6-311G(d) is used, revised numerical values must be used for qualification [39]. The marginal electrophiles have ω < 0.58 eV, moderate electrophiles have ω = (0.58 eV ÷ 0.97 eV), for strong electrophiles ω = (0.97 eV ÷ 2.38 eV), and ω values equal to or greater than 2.38 eV qualify a super electrophilic species. For nucleophilicity, the intervals are weak N < 1.92 eV; moderate N = (1.92 eV ÷ 2.95 eV); strong N = (2.95 eV ÷ 3.97 eV); and super nucleophilic N ≥ 3.97 eV [39]. Thus, **1**, **2c**, **6** are strong electrophiles, while **2a**,**b** are moderately electrophilic. The alkene compounds **1**, **6** are weakly nucleophilic; **2a**,**c** are moderate nucleophiles; and **2b** is a strong nucleophile.

According to global electro- and nucleophilicities, in the reactions, ethene **1** will act as the electrophile, while nitrile-*N*-oxides **2a**–**c** will act as the nucleophiles.

To characterize the reactivity of the substrates further, we computed the electrophilic and nucleophilic *Parr* functions for all of the substrates and the unsilylated alkene model. As the electronic properties of the compounds **1**, **2a**–**c**, and **6** are described at *ω*B97XD/6-311G(d) for the first time, both the electrophilic (P^+^_k_) and nucleophilic (P^−^_k_) *Parr* function values were computed.

The local electrophilicity (ω_k_) and nucleophilicity (N_k_) values for reactive sites, together with the corresponding values of the *Parr* function, are given in Table 2 and Table 3 below.

There is a small change in the Parr function between **1** and **6**. In both compounds, C5 is the more electrophilic center, while in the compounds **2a**–**c**, the more nucleophilic center is the oxygen atom. The most polarized nitrile-*N*-oxide moiety appears in compound **2b**.

**Figure 1:**
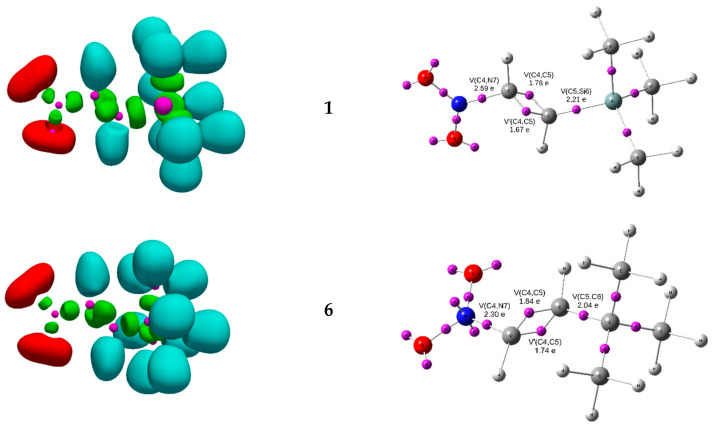
To the left: topology of the ELF function. Monosynaptic valence basins given in red, disynaptic in green, protonated basins in cyan, and core basins in magenta. To the right: positions of ELF attractors (in magenta) and populations of significant basins (isovalue = 0.75). Results for compounds **1** and **6** as computed via *ω*B97XD/6-311G(d).

**Figure 2:**
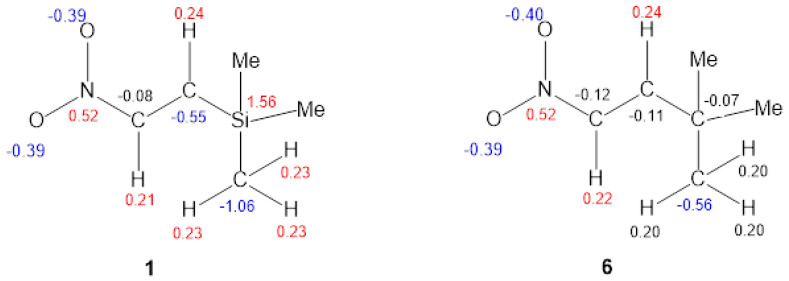
Natural charges of atoms (in electrons) in compounds **1** and **6** as computed at the ground state in gaseous phase (*ω*B97XD/6-311G(d)); charges > 0.2 e are given in red, charges < −0.2 are given in blue, and other charges are given in black. Methyl groups within XMe_3_ (X = Si,C) substituents are equivalent; thus, only one is described in detail.

To better understand the electronic structure of both compounds, they were characterized by ELF and NPA (Figure 1 and Figure 2).

Both alkenes (**1**, **6**) have the same number of disynaptic ELF attractors; the population of the disynaptic C4-C5 basin in **1** is 3.45 e, while in compound **6** it equates to 3.58 e (Figure 1), rendering the bonds virtually the same. There is, however, a strong difference between both **1** and **6** when it comes to the charges of the carbon atom bonded with the XMe_3_ group (X = Si,C) (Figure 2). The one in the t-Bu analogue has a negligible charge of −0.11 e, while the trimethylsilyl analogue has a negative charge of −0.55 e. Charge differences within the molecules suggest a push–pull effect.

The push–pull effect has a great impact on the reactivity of alkene-type compounds [40]; thus, compound **1** was screened for its effect in hope that the effect will explain the unexpected polarization of the C4-C5 bond in alkene (**1**), visible in the NPA results, as compared to alkene **6**. We have not found reports on the analysis of the push–pull effect via ELF. So, a new method is hereby proposed.

**Scheme 7:**
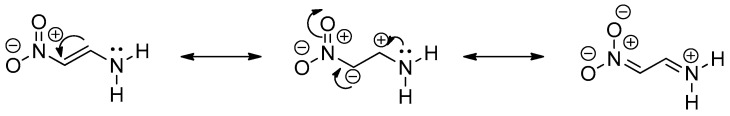
Resonance structures proposed by Rattananakin et al. for 2-amino-1-nitroethene [41].

The push–pull effect has two constituents: resonant and inductive [41]. In an alkene push–pull system, the C=C bond changes nature and becomes more reminiscent of a single bond; the stronger the effect, the less π electrons populate the bond [42]. Rattananakin et al. in 2007 [41] proposed three possible resonant structures explaining the push–pull system (Scheme 7).

In our example **1**, the electron donating group would be the SiMe_3_ moiety. Thus, the resonance effect will be nonexistent; only the inductive constituent remains. To quantify the inductive effect of a substituent, the F constant can be used [43].

In Table 4, the populations of the C1-C2 bonding region computed via ELF are given, as well as significant bond lengths.

Taking into consideration that the t-Bu group has a slightly stronger electron donating inductive effect (F_t-Bu_ = −0.02) than the SiMe_3_ (F_SiMe3_ = −0.01) group (F_NO2_ = 0.65), and that the C-Si bonds are longer than the C-C bonds, we conclude that there is no push–pull effect present neither in alkene **1** nor **6**, and that the changes in reactivity, shown in CDFT, most likely stem from geometrical differences between the compounds.

**Figure 3:**
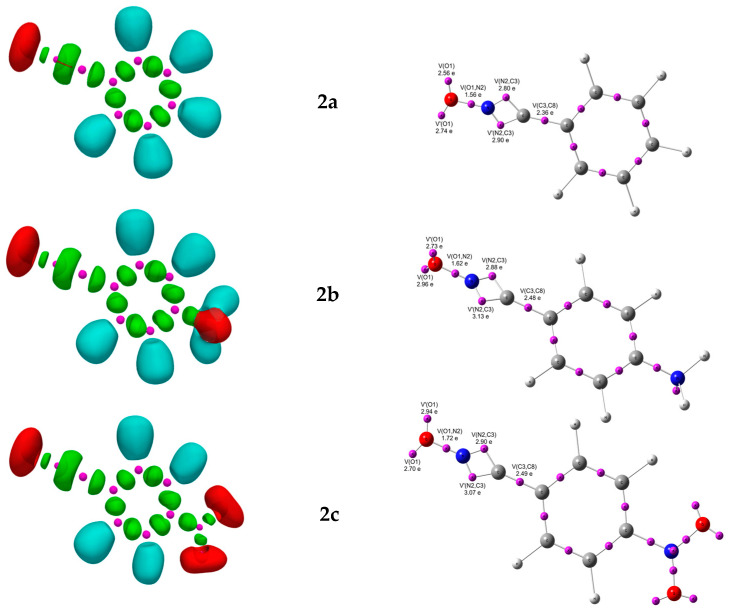
To the left: topology of the ELF function. Monosynaptic valence basins given in red, disynaptic in green, protonated basins in cyan, and core basins in magenta. To the right: positions of ELF attractors (in magenta) and populations of significant basins (isovalue = 0.75). Results for *N*-oxides **2a**–**c** as computed via *ω*B97XD/6-311G(d).

**Figure 4:**
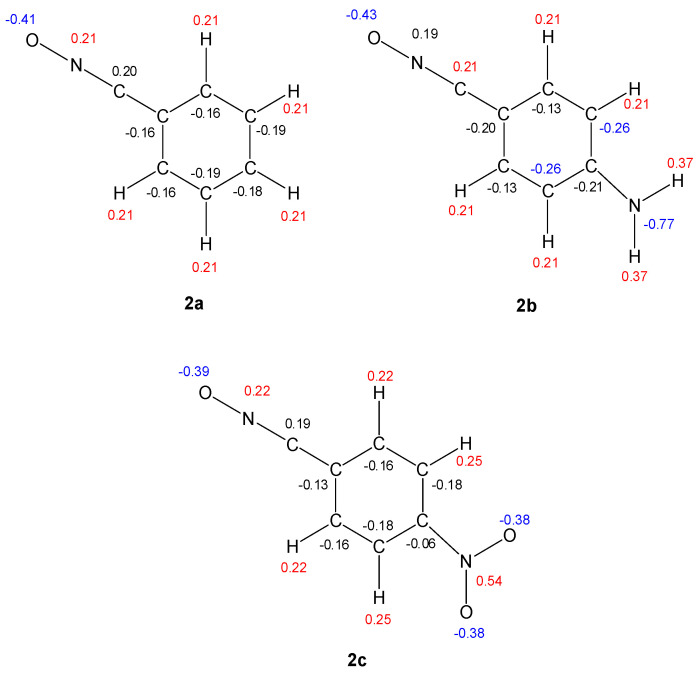
Natural charges of atoms (in electrons) in compounds **2a**–**c** as computed at the ground state in gaseous phase (*ω*B97XD/6-311G(d)); charges > 0.2 e are given in red, charges < −0.2 are given in blue, and other charges are given in black.

Nucleophilic agents (**2a**–**c**) were also studied with ELF and NPA (Figure 3 and Figure 4).

The CNO moiety of *N*-oxides **2a**–**c** shows three slightly underpopulated lone pairs on the oxygen atom (Figure 3) and an underpopulated C-N single bond (NPA analysis shows that there is ionic component to the C-N bonds—Figure 4). Oxides **2a** and **2c** show a slightly underpopulated C≡N bond, while oxide **2b** has the bond fully populated. In all the oxides, disynaptic basin N2,C3 shows rotational symmetry, for which the axis covers a line that stretches through C-N-O. The CNO group is completely linear. The C3-C8 bond is an overpopulated (excess of 0.36–0.49 e) single bond, suggesting that the CNO moiety is somewhat conjugated with the phenyl aromatic system.

The red strip visible on the N2-C3 ELF isovalue surface of *N*-oxide **2a** in Figure 3 is an artifact, resulting from the Topmod symmetry-based computation acceleration, and there is no monosynaptic attractor in the volume between the C3 and N2 atoms.

**Figure 5:**
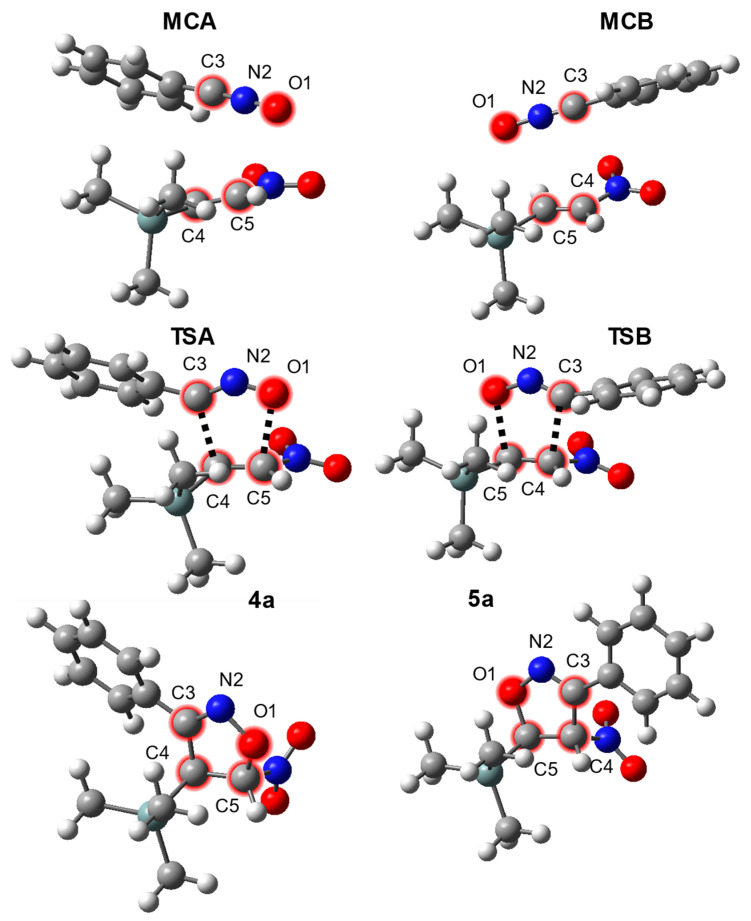
Views of critical structures for the (3+2) cycloaddition reaction between E-2-(trimethylsilyl)-1-nitroethene (**1**) and benzonitrile *N*-oxide (**2a**) in the CCl_4_ solution in light of the *ω*B97XD/6-311+G(d) (PCM) calculations.

In the next step of our research, we explored the reaction profiles for both of the possible 32CA channels in the carbon tetrachloride environment used under the experimental condition as solvents. For this purpose, the DFT calculation at the wb97xd/6-311+G(d) (PCM) level of theory was used. Within these considerations, model 32CA of E-2-(trimethylsilyl)-1-nitroethene (**1**) with benzonitrile *N*-oxide (**2a**) was analyzed. It was found that the nature of both of the considered profiles is very similar. In particular, independently of the 32CA channel, two critical points were detected and verified between the areas of individual reagents and respective products. The graduate reduction in distance between reaction components leads, in the initial stage, to the formation of a respective pre-reaction complex (**MCA** for path **A** and **MCB** for path **B**, respectively). This process is barrierless and associated with a reduction in the enthalpy of the reaction system by a few kcal/mol (Table 5). It should be underlined that at the same time, the entropy of the reaction systems is substantially reduced. As a consequence, the *Gibbs* free energies of the formation of both MCs are positive. This excludes the possibility of the existence of a pre-reaction complex as relative thermodynamic stable intermediates. Within MCs, the substructures of reagents adopt the orientation that determined the further conversion along the respective, regioisomeric reaction channel (Figure 5). So, these intermediates should be treated as orientation complexes [44,45,46,47]. It is important, however, to ensure that no new sigma bonds were not formed at this stage. The key interatomic distances exist in the range of 3–3.7 Å (Table 6) (evidently beyond the area typical for C-C and C-O distances in transition states). In the framework, the electron density transfer between substructures is also not observed (GEDT = 0 e) (Table 6). So, localized intermediates are not charge–transfer complexes [48]. In general, similar types of intermediates were identified experimentally regarding 32CA of ozone with ethene and ethyne [49,50].

The further conversion of the reaction system along the intrinsic reaction coordinate leads to the formation of the transition state independently of the 32CA channel. These are transition states **TSA** and **TSB** for channels **A** and **B**, respectively. The formation of the transition state is associated with an increase in the enthalpy of the reaction system by 10.4 kcal/mol and 10.8 kcal/mol for **TSA** and **TSB**, respectively (Table 5). Including the entropy factor for the calculation of the activation barrier stimulates values of the Gibbs free energy of the activation 24.4–24.8 kcal/mol. So, both theoretically possible 32CA paths are allowed from the kinetic point of view. This undermine the proposed earlier postulate about 3-phenyl-4-trimethylsillyl-5-nitroisoxazoline **4a** as the only possible adduct in this cycloaddition reaction.

The localized TS exhibits a nature that is typical for the single-step 32CA with the participation of linear-type TACs [19,51,52,53,54]. Within these transition states, two new sigma bonds are formed. These bonds exhibit, however, a different degree of development. The new bond is always formed slightly faster at the b-carbon atom from the nitrovinyl > C=C(NO_2_) moiety.

Both optimized transition states exhibit the transfer of the electron density (GEDT) between substructures. Values of the GEDT indices are typical for the polar but one-step 32CAs [55,56,57].

**Figure 6:**
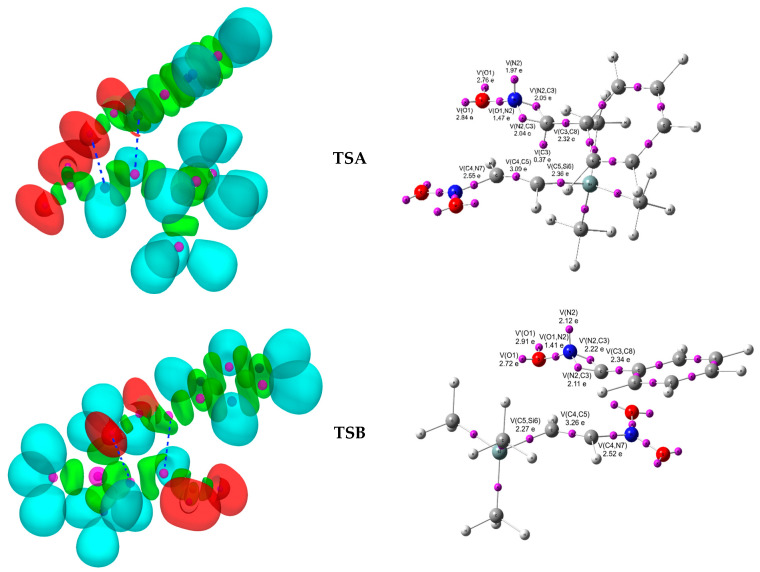
To the left: topology of the ELF function. Monosynaptic valence basins given in red, disynaptic in green, protonated basins in cyan, and core basins in magenta (isovalue = 0.75). The blue dashed lines signify which atoms will bond if the TS transforms to the products. To the right: positions of ELF attractors (*ω*B97XD/6-311G(d) PCM-CCl_4_.

**Figure 7:**
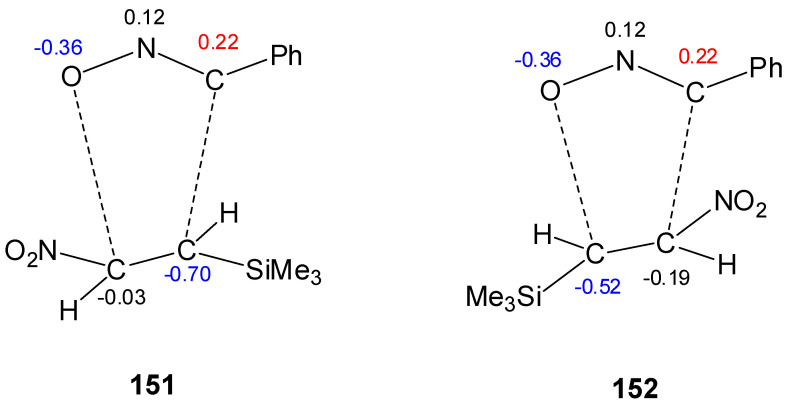
Natural charges of atoms (in electrons) in **TSA** and **TSB** as computed at the ground state in gaseous phase (*ω*B97XD/6-311G(d), PCM CCl_4_); charges > 0.2 e are given in red, charges < −0.2 are given in blue, and other charges are given in black.

The electronic structure of the TS-s was characterized using NPA and ELF. In the case of NPA (Figure 6 and Figure 7), there was no significant change in the charges between the substrates and TS outside of the reacting site; thus, the phenyl, trimethylsilyl, and nitro groups are not broken down.

In both transition states, the depopulation of the C4=C5 double bond compared to the substrates is visible. Also, the N2 atom accepts one electron pair, changing the C3≡N2 triple bond into a double one. During the process, the CNO moiety loses its linearity (Figure 6). Bonds that will not enter the five-membered ring, when the TS turns into a product, do not change significantly when compared to substrates. There seems to be an electrostatic repulsion between the O1 and C5 atoms, yet the repulsion is stronger in **TSB**. Considering that in the TSs both C3≡N2 triple bonds and C4=C5 double bonds are depopulating, and that there is a significant difference in electrostatic interactions between the C3-C4 atom pair (attraction) and O1-C5 atom pair (repulsion), we propose that the TSs stand for a single-step asynchronic mechanism.

The IRC calculations confirmed without any doubt that the **TSA** is a single-transition structure along path **A**, and similarly, **TSB** is a single-transition structure along path **B**. All attempts to optimize the hypothetical zwitterionic intermediates on the basis of the interactions between E-2-(trimethylsilyl)-1-nitroethene (**1**) and benzonitrile *N*-oxide (**2a**) were not successful. So, the polar but one-step mechanism of model 32CA **1** + **2a** is evident in light of this DFT computational study.

In the framework of the extended study, we examined the same reaction in more polar solvents (acetone, nitromethane—ε = 20.493 and ε = 36.562, respectively [58]). It was found that the increase in the polarity of the reaction environment stimulated the increase in the enthalpy of the activation on both of the considered cycloaddition paths. These changes are not, however, spectacular and do not exceed 1.5 kcal/mol. Rather weak is also the solvent effect in the case of the key geometrical parameters of transition states. In particular, the asynchronicity of both TSs is only slightly higher in the nitromethane solution in comparison to the analogous structures in carbon tetrachloride. The respective GEDT values are also very similar.

Next, we analyzed the substituent effect on the reaction course. For this purpose, we examined the analogous cycloaddition reaction with the participation of nitrile oxides functionalized by the amino (s = −0.66 [43]) and nitro (s = 0.78 [43]) groups.

**Scheme 8:**
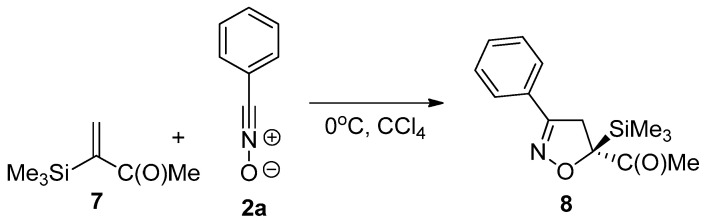
Experimentally observed course of the (3+2) cycloaddition reaction between 1-(trimethylsilyl)-1-acetylethene (**7**) and benzonitrile *N*-oxide (**2a**).

Lastly, we performed the exploration of energy profiles for the analogous reaction with the participation of the 1-(trimethylsilyl)-1-acetylethene (**7**) (Scheme 8). In this reaction, the primary cycloaddition product was isolated and identified as the 5,5-disubstituted isooxazoline derivative. The same regioselectivity is suggested in our *ω*B97XD/6-311+G(d) (PCM) computational study. This conclusion supports that *ω*B97XD/6-311+G(d) (PCM) offers the correct view of the selectivity of the title reactions.

## 3. Computational Details

The DFT computations were performed using the *ω*B97XD/6-311+G(d) level of theory implemented in the Gaussian package [59,60]. The computational PlGrid infrastructure at the polish “Cyfronet” center was utilized. A similar computational level and the methodology have already been successfully applied very recently to explore the mechanistic aspects of various cycloaddition processes [52,61,62,63]. All localized stationary points were verified on the basis of the full vibrational analysis. We found that starting molecules, pre-reaction complexes, and products had positive Hessian matrices. On the other hand, all of the optimized transition states (**TSs**) exhibited one negative eigenvalue in their Hessian matrices. Next, the IRC (intrinsic reaction coordinate) trajectories computed for all TSs confirmed, without doubt, the postulated nature and role within the energy profile. The presence of solvents (carbon tetrachloride, acetone, nitromethane) in the reaction environment was included using the IEFPCM (Integral Equation Formalism Polarizable Continuum Model) algorithm [64]. Calculations of all critical structures were performed at a temperature of T = 298 K and pressure of p = 1 atm. The results are collected in Table 5 and Table 6.

The global electron density transfer (GEDT) [65] within critical structures was estimated using the following formula:

GEDT = −ΣqA

where qA is the net charge, and the sum is taken over all of the atoms of nitroalkene.

The global and local electronic properties of the reactants were estimated using equations recommended by *Parr* and *Domingo* [66,67]. In particular, the electronic chemical potentials (µ) and chemical hardness (η) were evaluated in terms of the one-electron energies of the frontier molecular orbitals (HOMO and LUMO) using the following equations:

μ ≈ (E_HOMO_ + E_LUMO_)/2  η ≈ E_LUMO_ − E_HOMO_

It should be mentioned at this point that orbital energies computed at the DFT level have some errors and starting point dependence. For the homogenous series of reagents, these errors are, however, similar and, in general analysis, exhibit a non-important role [68]. The values of µ and η were then used to calculate the global electrophilicity index (ω) using the following formula:

ω = μ^2^/2η

Global nucleophilicity (N) [69] was expressed using the following equation:

N = E_HOMO_ − E_HOMO (tetracyanoethene)_

The local electrophilicity (ω_k_) at atom *k* was calculated by projecting the index ω onto any reaction center *k* in the molecule using *Parr* functions P^+^_k_:

ω_k_ = P^+^_k_·ω

The local nucleophilicity (N_k_) condensed to atom *k* was calculated using global nucleophilicity N and *Parr* functions P^−^_k_ [70] according to the following formula:

N_k_ = P^−^_k_·N

The results are summarized in Table 1.

## 4. Conclusions

This *ω*B97XD/6-311+G(d) (PCM) computational study sheds light on the regioselectivity and molecular mechanism of the (3+2) cycloaddition reaction between E-2-(trimethylsilyl)-1-nitroethene and arylonitrile *N*-oxides. Our results undermine the proposed earlier postulate about 3-phenyl-4-(trimethylsilyl)-5-nitroisoxazoline **4a** as the only possible adduct in this cycloaddition reaction. The obtained parameters of the activation allow for the reaction course to be theoretically possible via both regioisomeric cycloaddition channels. Next, the DFT simulations exclude the possibility of the formation of the zwitterionic intermediate along the reaction course. The electronic nature of both optimized TSs was identified using the ELF technique. Additionally, the role of the global and local polar interactions was described on the basis of the CDFT descriptors. Lastly, the exploration of the solvent and substituent effects show that the proposed mechanism can be treated as general for some cycloaddition groups.

## Figures and Tables

**Table 1:** Chemical potential (µ), chemical hardness (η), global electrophilicity (ω), and nucleophilicity (N) of compounds **1**, **2a**–**c**, and **6**, as computed via *ω*B97XD/6-311G(d); all values are expressed in eV.

Compound	μ	η	ω	N
**1**	−5.45	9.74	1.53	1.07
**6**	−5.34	9.93	1.44	1.09
**2a**	−4.10	8.78	0.96	2.91
**2b**	−3.39	8.47	0.68	3.77
**2c**	−5.24	7.80	1.76	2.26

**Table 2:** *Parr* function values and local electrophilicity and nucleophilicity for significant atoms in alkenes **1** and **6**.

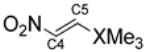	**Compound**	**X**	**ω_C5_ (P^+^_C5_)**	**ω_C4_ (P^+^_C4_)**	**N_C5_ (P^−^_C5_)**	**N_C4_ (P^−^_C4_)**
	**1**	Si	0.58 (0.38)	0.11 (0.07)	0.08 (0.08)	0.03 (0.02)
	**6**	C	0.59 (0.41)	−0.01 (0.00)	−0.02 (−0.02)	0.00 (0.00)

**Table 3:** *Parr* function values and local electrophilicity and nucleophilicity for significant atoms in N-nitrile oxides **2a**–**c**.

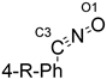	**Compound**	**R**	**ω_C3_ (P^+^_C3_)**	**ω_O1_ (P^+^_O1_)**	**N_C3_ (P^−^_C3_)**	**N_O1_ (P^−^_O1_)**
	**2a**	H	−0.05 (−0.05)	0.08 (0.08)	0.07 (0.02)	1.46 (0.50)
	**2b**	NH_2_	0.01 (0.02)	−0.01 (−0.02)	−0.38 (−0.10)	1.24 (0.33)
	**2c**	NO_2_	−0.12 (−0.07)	0.10 (0.06)	0.15 (0.07)	1.20 (0.53)

**Table 4:** Populations of bonding regions and bond lengths in compounds **1** and **6**, *ω*B97XD/6-311G(d).

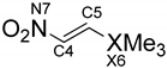	**Compound**	**X**	**C4-C5 population ^1^**	**l_C4-C5_ ^2^**	**l_C4-N7_ ^2^**	**l_C5-X6_ ^2^**	**l_X6-CMe_ ^3^**
	**1**	Si	3.45	1.32	1.47	1.88	1.87
	**6**	C	3.58	1.32	1.46	1.50	1.53

^1^ Population of C4-C5 ELF bonding volume in electrons; ^2^ bond length in Å; ^3^ bond length in Å between atom X and a carbon atom of methyl group in XMe_3_ substituent.

**Table 5:** Kinetic and thermodynamic parameters for the (3+2) cycloaddition reactions between (trimethylsilyl)-substituted alkenes (**1** and **7**) and nitrile *N*-oxides (**2a**–**c**) in light of the *ω*B97XD/6-311+G(d) (PCM) calculations (ΔH, ΔG are in kcal/mol; ΔS are in cal/molK).

Reaction	Solvent	Path	Transition	ΔH	ΔS	ΔG
**1** + **2a**	CCl_4_	**A**	**1** + **2a→MCA**	−6.0	−34.1	4.2
			**1** + **2a→TSA**	10.4	−48.4	24.8
			**1** + **2a→4a**	−46.4	−51.3	−31.1
		**B**	**1** + **2a→MCB**	−4.6	−33.1	5.3
			**1** + **2a→TSB**	10.8	−45.5	24.4
			**1** + **2a→5a**	−38.7	−48.4	−24.3
	Acetone	**A**	**1** + **2a→MCA**	−5.6	−33.2	4.3
			**1** + **2a→TSA**	11.1	−48.6	25.6
			**1** + **2a→4a**	−46.2	−51.0	−30.9
		**B**	**1** + **2a→MCB**	−5.1	−41.6	7.3
			**1** + **2a→TSB**	12.1	−46.0	25.8
			**1** + **2a→5a**	−37.5	−49.4	−22.8
		**A**	**1** + **2a→MCA**	−5.6	−33.4	4.4
	MeNO_2_		**1** + **2a→TSA**	11.1	−48.6	25.6
			**1** + **2a→4a**	−46.2	−50.9	−31.0
		**B**	**1** + **2a→MCB**	−5.1	−41.6	7.3
			**1** + **2a→TSB**	12.2	−45.8	25.8
			**1** + **2a→5a**	−37.4	−49.5	−22.7
**1** + **2b**	CCl_4_	**A**	**1** + **2b→MCA**	−6.6	−35.2	3.9
			**1** + **2b→TSA**	9.7	−48.0	24.0
			**1** + **2b→4b**	−46.2	−49.3	−31.5
		**B**	**1** + **2b→MCB**	−4.9	−34.0	5.2
			**1** + **2b→TSB**	9.5	−45.5	23.1
			**1** + **2b→5b**	−38.4	−49.0	−23.8
**1** + **2c**	CCl_4_	**A**	**1** + **2c→MCA**	−5.6	−38.8	6.0
			**1** + **2c→TSA**	10.9	−48.6	25.4
			**1** + **2c→4c**	−47.2	−51.6	−31.8
		**B**	**1** + **2c→MCB**	−5.4	−37.8	5.9
			**1** + **2c→TSB**	11.7	−49.2	26.3
			**1** + **2c→5c**	−39.9	−49.6	−25.1
**2a** + **7**	CCl_4_	**A**	**2a** + **7→MCA**	−5.9	−35.0	4.6
			**2a** + **7→TSA**	10.6	−42.3	23.2
			**2a** + **7→8**	−44.9	−48.5	−30.5
		**B**	**2a** + **7→MCA**	−6.5	−36.1	4.3
			**2a** + **7→TSA**	10.6	−46.5	24.5
			**2a** + **7→9**	−41.5	−53.0	−25.7

**Table 6:** Key parameters of critical structures for the (3+2) cycloaddition reactions between (trimethylsilyl)-substituted alkenes (**1** and **7**) and nitrile *N*-oxides (**2a**–**c**) in light of the *ω*B97XD/6-311+G(d) (PCM) calculations.

Solvent	Path	Structure	Interatomic Distances [Å]					GEDT
			O1-N2	N2-C3	C3-C4	C4-C5	C5-O1	[e]
CCl_4_	**A**	**1**				1.324		
		**2a**	1.210	1.154				
		**MCA**	1.209	1.154	3.604	1.324	3.671	
		**TSA**	1.218	1.203	2.126	1.366	2.269	0.25
		**4a**	1.415	1.278	1.501	1.521	1.379	
	**B**	**MCB**	1.213	1.153	3.429	1.325	3.057	
		**TSB**	1.233	1.200	2.204	1.372	2.143	0.17
		**5a**	1.357	1.279	1.510	1.519	1.459	
Acetone	**A**	**1**				1.325		
		**2a**	1.217	1.152				
		**MCA**	1.215	1.153	3.670	1.325	3.778	
		**TSA**	1.221	1.203	2.111	1.367	2.282	0.27
		**4a**	1.420	1.279	1.499	1.519	1.379	
	**B**	**MCB**	1.216	1.153	4.431	1.325	4.928	
		**TSB**	1.237	1.199	2.211	1.372	2.136	0.16
		**5a**	1.360	1.280	1.511	1.518	1.460	
MeNO_2_	**A**	**1**				1.325		
		**2a**	1.217	1.152				
		**MCA**	1.216	1.152	3.671	1.325	3.776	
		**TSA**	1.221	1.203	2.110	1.367	2.284	0.27
		**4a**	1.420	1.279	1.498	1.519	1.379	
	**B**	**MCB**	1.216	1.153	4.430	1.325	4.927	
		**TSB**	1.237	1.199	2.212	1.373	2.135	0.16
		**5a**	1.360	1.280	1.511	1.518	1.461	
CCl_4_	**A**	**2b**	1.215	1.154				
		**MCA**	1.214	1.155	3.604	1.324	3.706	
		**TSA**	1.223	1.205	2.129	1.366	2.244	0.20
		**4b**	1.420	1.280	1.503	1.520	1.376	
	**B**	**MCB**	1.217	1.153	3.410	1.325	3.043	
		**TSB**	1.239	1.203	2.225	1.373	2.100	0.21
		**5b**	1.363	1.280	1.512	1.518	1.457	
CCl_4_	**A**	**2c**	1.204	1.155				
		**MCA**	1.203	1.155	3.631	1.324	3.726	
		**TSA**	1.213	1.203	2.133	1.365	2.287	0.31
		**4c**	1.407	1.278	1.500	1.521	1.382	
	**B**	**MCB**	1.207	1.154	3.442	1.325	3.120	
		**TSB**	1.227	1.199	2.196	1.370	2.174	0.15
		**5c**	1.348	1.462	1.518	1.510	1.280	
CCl_4_	**A**	**7**				1.338		
		**MC**	1.210	1.154	3.449	1.337	3.958	
		**TS**	1.217	1.202	2.137	1.375	2.417	0.24
		**8**	1.388	1.278	1.508	1.530	1.450	
	**B**	**MC**	1.214	1.153	3.594	1.338	3.462	
		**TS**	1.232	1.202	2.293	1.385	2.110	0.05
		**9**	1.380	1.278	1.519	1.547	1.437	

## Data Availability

The original contributions presented in this study are included in the article. Further inquiries can be directed to the corresponding author.
